# Mixed mycotic infection in an immunocompromised host with advanced HIV diagnosed with the assistance of a Karius test

**DOI:** 10.1016/j.mmcr.2026.100761

**Published:** 2026-01-22

**Authors:** Elise Hyser, Shirisha Pasula

**Affiliations:** Division of Infectious Diseases, Department of Medicine, UCSF Fresno, USA

**Keywords:** Pneumocystis jirovecii pneumonia (PJP), Histoplasma capsulatum, Coccidioides posadasii, Mixed mycotic infection, Karius test

## Abstract

We report the case of a 35-year-old female with advanced HIV diagnosed with a mixed mycotic infection. Diagnosis was assisted by Karius test. She was admitted with postpartum psychosis and developed fever and acute hypoxic respiratory failure initially thought to be from a bacterial hospital-acquired pneumonia. Due to lack of sufficient improvement on appropriate antibiotics, a broad infectious workup was sent. A Karius test was performed early during the workup. It returned positive for *Pneumocystis jirovecii*, *Coccidioides posadasii*, and *Histoplasma capsulatum*. Eventually a standard work up returned with a positive bronchoalveolar lavage (BAL) *Pneumocystis jirovecii pneumonia (PJP)* test by direct fluorescent antibody (DFA), positive *Histoplasma* urine antigen, and positive serum coccidioidal titer of 1:16, corroborating the diagnosis of mixed mycotic infection. She was treated with a 21-day course of trimethoprim-sulfamethoxazole followed by primaquine and clindamycin for *PJP*. She clinically improved with over 3 weeks of IV amphotericin B to cover for histoplasmosis and coccidioidomycosis, which was later de-escalated to oral itraconazole. She was instructed to follow up in clinic where bictegravir/emtricitabine/tenofovir alafenamide was initiated. This case highlights the utility of building a broad differential diagnosis as mixed mycotic infections can coexist in an immunocompromised host, and of utilizing a Karius test early in the course of illness in an immunocompromised patient to expedite a diagnosis. This case also demonstrates the need to recognize the changing geographic distribution of fungal infections.

## 1 Introduction

While mixed mycotic infections are uncommon, they have a proclivity for severely immunocompromised hosts like transplant recipients and advanced HIV patients.

*Pneumocystis jirovecii* is a ubiquitous fungus that can infect immunocompromised hosts [1]. *PJP* cases are diagnosed predominantly in patients with advanced HIV possessing a CD4 count <200 cells/mm^3 1^. Prior to the advent of *PJP* prophylaxis, *PJP* was diagnosed in 70–80 % of patients who had advanced HIV [1]. Since the widespread utilization of *PJP* prophylaxis and antiretroviral therapy, the incidence of *PJP* has notably decreased such that most cases are diagnosed in patients not receiving care for HIV and patients who are profoundly immunosuppressed [1]. It commonly presents with a subacute course of fever, dyspnea, non-productive cough, and pleuritic chest pain [1]. Hypoxemia can be mild, moderate, or severe. Elevated LDH and elevated beta D glucan are frequently observed although nonspecific [1]. Diffuse ground-glass opacities are typically demonstrated, but atypical imaging findings including nodules, upper lobe localized infection, cysts, and intrathoracic lymphadenopathy can occur as well [1]. Histopathologic or cytologic visualization of *Pneumocystis jirovecii* in tissue, BAL, or induced sputum facilitates the diagnosis [1]. In fact, a positive Beta D glucan is analyzed in conjunction with a *Pneumocystis*-specific assay, such as histochemical or molecular detection via PCR, and compatible imaging findings to solidify the diagnosis of PJP [2]. Bronchoscopy with bronchoalveolar lavage is associated with a >90 % diagnostic yield for *PJP* and is thus a critical laboratory component of the diagnosis [2]. A 21-day course of trimethoprim-sulfamethoxazole is the preferred therapy.

*Histoplasma capsulatum* is a dimorphic fungus endemic to the central and south-central U.S. [3]. Among HIV patients, a CD4 count <150/mm^3^ raises the risk of symptomatic infection [3]. Decreased cellular immunity, which occurs in advanced HIV, can reactivate an infection acquired years before [3]. Typical symptoms at the onset of infection include fevers, chills, cough, dyspnea, and chest pain [4]. Fifty percent of patients experience cough and dyspnea, while patients with advanced HIV who have the clinical picture of disseminated histoplasmosis experience fatigue, fevers, weight loss, and hepatosplenomegaly [3]. Dissemination to the CNS occurs in 20 % or less of patients diagnosed with histoplasmosis [3]. Chest imaging can demonstrate diffuse patchy nodular opacities with hilar and mediastinal lymphadenopathy [5]. Identification of *Histoplasma* antigen in the blood or urine facilitates the diagnosis [3]. The treatment is liposomal amphotericin B induction for at least 2 weeks or until clinical improvement, followed by itraconazole maintenance for at least one year depending on infection severity [4].

*Coccidioides posadasii* is a dimorphic fungus endemic to areas of the southwestern United States including California, Nevada, Arizona, New Mexico, and Texas [6,7]. The number of cases more than doubled between 2014 (when the case number was 8232) and 2019 (when the case number was 20,003) [6]. California and Arizona carry over 95 % of reported cases of coccidioidomycosis nationally [6]. Heterogeneous symptoms and imaging findings occur with pulmonary coccidioidomycosis, and some cases mimic community acquired pneumonia [6]. The enzyme immunoassay can identify *Coccidioides* IgM and IgG antibodies [6]. *Coccidioides* immunodiffusion and complement fixation provide diagnostic confirmation [6]. Treatment is based on disease severity, clinical history, and comorbidities [6]. Fluconazole is often utilized, although other azole antifungal agents can be given if it is not tolerated [6]. In severe acute pulmonary coccidioidomycosis, liposomal amphotericin B is administered. For cases in which coccidioidomycosis disseminates to the CNS, high dose azole antifungals such as fluconazole are utilized [7]. Complications associated with CNS coccidioidomycosis carry high morbidity and mortality and include hydrocephalus, arachnoiditis, stroke, and brain abscess [7].

While *PJP*, histoplasmosis, and coccidioidomycosis have overlapping clinical syndromes, they do not typically co-infect the same host. Here we present a case of an advanced HIV patient presenting with fever, chills and lung infiltrates diagnosed with concurrent fungal infections: *PJP*, histoplasmosis and coccidioidomycosis.

This case demonstrates the importance of formulating a broad differential diagnosis as mixed mycotic infections can coinfect an immunocompromised host. The case also reveals that a Karius test can facilitate the diagnosis of complicated co-infections afflicting an immunocompromised host. Since mixed mycotic infections lead to atypical presentations, fungal detections that are above reporting thresholds on Karius test can serve as an adjunct in the diagnosis. The Karius test can be used in conjunction with clinical features, fungal serologies, culture data, historical information, and imaging findings to solidify the diagnosis. Additionally, the case generates discussion about the changing geographic distribution of fungal infections. Histoplasmosis is generally observed in the Mississippi and Ohio River valleys, but climate change and other anthropogenic factors have altered the geographic distribution of the infection [3,8].

## 2 Case

The patient is a 35-year-old female with advanced HIV presenting on admission (day 0) with a 5-day history of bizarre behavior including paranoid delusions. She endorsed auditory hallucinations that someone was attempting to kill her. She denied any concomitant headaches, neck pain, vision changes, or focal weakness. She did not have any history of intravenous drug use or alcohol abuse.

She was diagnosed with HIV one year prior to admission and briefly followed up at the local HIV clinic. She refused to take HIV medications due to concerns related to side effects and preferred herbal therapies to antiretroviral medications. Her CD4 count on day −3 was 74 cells/μL and a recent HIV viral load on day −21 was 789,000 copies/mL. She has a history of verbal and physical partner violence. She had a normal spontaneous vaginal delivery one year prior to admission complicated by late prenatal care. On day −21 she had another vaginal delivery with newborn loss. She is originally from California and currently lives in the Central Valley. Travel history elicits that she visited Arizona and Virginia one year prior to admission. She travelled to Delaware years before her admission.

On day 0, her initial vitals included a temperature of 36.4 °C, heart rate of 92 beats per minute, respiratory rate of 16 breaths per minute, O_2_ saturation of 99 % on room air, and blood pressure of 113/81. Physical exam demonstrated that she was alert and oriented to person, place, and time with no focal neurological deficits. Auditory hallucinations were noted. She had clear lungs, an unremarkable cardiac examination, normal abdominal examination, and no rashes.

She underwent a comprehensive workup to exclude infection as the source of her altered mental status (see Table 1, Table 2). Her white blood cell count was 24 (reference range 4–11 x 10^3/μL), hemoglobin was 9.3 (reference range 12–16g/dL), and platelet count was 384 (reference range 140–440 x 10^3/μL). Kidney and liver function tests were unremarkable. Lumbar puncture showed a CSF cell count with differential within normal limits. Total nucleated cell count was 1. CSF protein was 35. CSF glucose was 186. CSF meningitis panel, CSF bacterial cultures, CSF *Coccidioides* PCR, CSF *Coccidioides* identification and complement fixation were negative. CSF human polyomavirus 2 (JC virus) was negative. Chest x-ray revealed mild right lower lobe infiltrates. Streptococcal urine antigen returned positive. Respiratory pathogen panel was negative. She was initially diagnosed with community acquired pneumonia. Ceftriaxone and azithromycin were given from day 0 to day 3, then antibiotics were de-escalated to cefpodoxime. However, she developed persistent fevers of 102–103 °F associated with rigors. Her cough and dyspnea persisted. Antimicrobial therapy was escalated to cefepime to provide empiric coverage of *Pseudomonas aeruginosa* due to concern for possible developing hospital acquired pneumonia.

**Table 1:** CBC and CMP on admission, at the time of diagnosis of the 3 fungal infections, and upon discharge.

Labs Collected	Labs on Admission	Labs at Diagnosis	Labs upon Discharge
**Hematology**			
WBC (reference range: 4-11 x 10^3/μL)	24	5	2.4
Hemoglobin (reference range: 12–16g/dl)	9.3	9.1	8.4
Platelet Count (reference range: 140-440 x 10^3/μL)	384	303	224
**Electrolytes**			
Na+ (reference range: 135–145mmol/L)	135	140	136
K+ (reference range: 3.5–5.3 mmol/L)	3.5	3.1	4.2
Cl- (reference range: 98–110 mmol/L)	105	108	105
**Kidney and Liver Function**			
Creatinine (reference range: 0.5–1.1 mg/dL)	0.6	0.8	0.7
ALT (reference range: 7–35 U/L)	6	12	50
AST (reference range: 8–40 U/L)	17	30	66

**Table 2:** HIV labs and further workup.

Labs Collected	
**HIV Labs**	
CD4 Count (reference range: 490–1740 cells/μL)	74
HIV Viral Load (copies/mL)	789,000
**Further Workup**	
Histoplasma Urine Ag (reference range: <0.2 ng/mL)	0.5
Coccidioides Serum Complement Fixation Titer	1:16
PJP by DFA from Bronchoalveolar Lavage	Positive
Aspergillus Serum Ag	Negative
Beta D glucan (reference range: <60pg/mL)	>500
Karius Test	PJP, *C. posadasii*, *H. capsulatum*, *E. coli*
Acid Fast Bacilli (AFB) stains from BAL	Negative
Quantiferon Gold	Negative

## 3 CT chest

**Figure:**
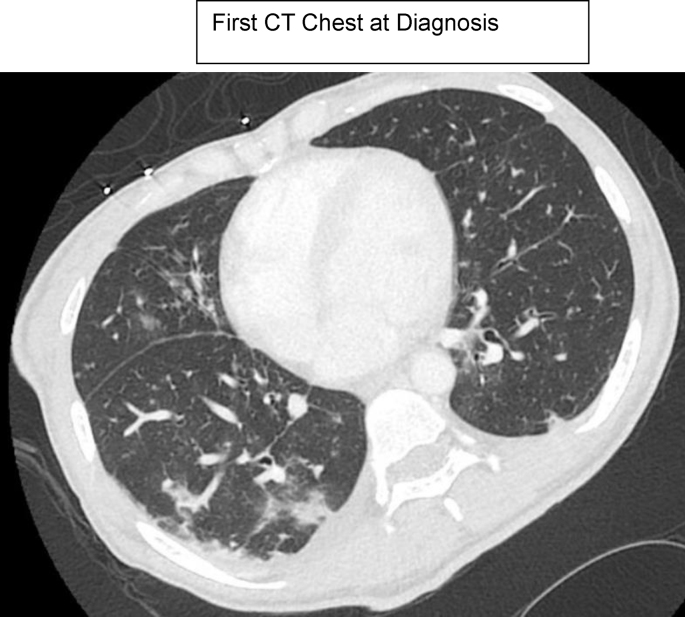

**Figure:**
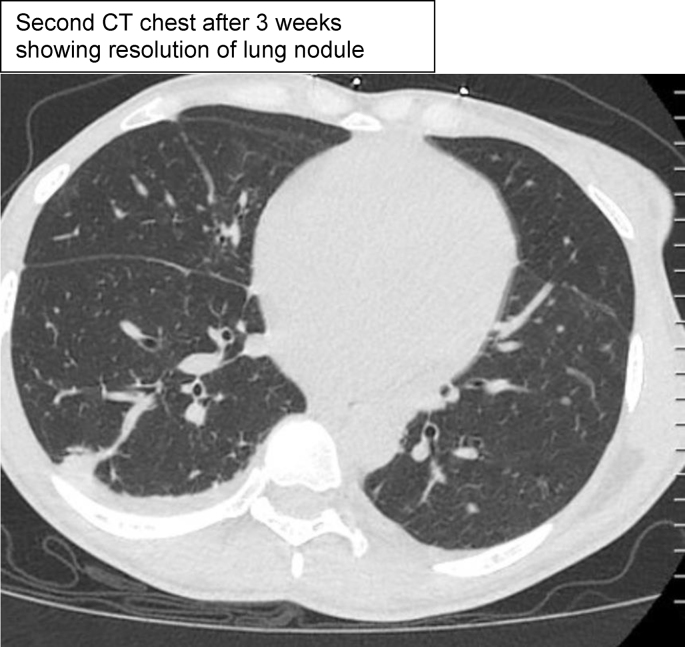

CT chest demonstrated multiple small nodular opacities in the right lower lobe and mediastinal lymphadenopathy. CT head and lumbar puncture were unremarkable. A Karius test was added due to her tenuous clinical status and returned positive for *PJP*, *Coccidioides posadasii*, *Histoplasma capsulatum*, and *Escherichia coli*. Later, *Histoplasma* urine antigen returned positive at a level of 0.5 (reference range <0.2ng/mL), *Coccidioides* serologies via enzyme immunoassay showed a + IgM, + IgG, and + CF, *Coccidioides* serum complement fixation titer returned at 1:16, *PJP* by DFA via BAL was positive, LDH was 305 (reference range 135–214 U/L), and Beta D glucan was >500 (reference range <60pg/mL). Aspergillus galactomannan antigen from the BAL (Platelia Aspergillus EIA kit from MiraVista Diagnostics) was 2.30 (reference range negative), but aspergillus serum antigen was negative, aspergillus was not isolated on cultures, and she did not have the clinical syndrome of aspergillosis. Cross-reactivity can be observed with aspergillus species. A Karius test was performed on the blood and ultimately detected high levels of *Pneumocystis jirovecii, Coccidioides posadasii,* and *Histoplasma capsulatum.*

Plasma microbial cell-free DNA (mcfDNA) metagenomic sequencing was utilized during the Karius test. Whole-blood samples were collected in K2-EDTA tubes. Plasma separated from cells (stable at ambient temperature for 96 hours and at −20 °C for 6 months) was sent to the Karius, Inc. clinical laboratory (Redwood City, CA), certified under the Clinical Laboratory Improvement Amendments of 1988 and accredited by the College of American Pathologists for plasma mcfDNA sequencing as previously described [9,10]. Sequencing data were analyzed using the Karius Test® bioinformatic pipeline version DC 4.1, which was designed to report mcfDNA from over 1000 microbes and provides the absolute plasma concentration of mcfDNA in molecules per 100 nL (MPHN) for each microbe detected [9,10]. As MPHN values from different microbes are not comparable, a reference interval determined from a study of 160 asymptomatic adults is provided in clinical result reports for comparison as an aid to interpretation.

Plasma mcfDNA detections: Three fungi mcfDNA (*Pneumocystis jirovecii,* 2602.1 MPHN; *Coccidioides posadasii*, 3014.3 MPHN; and *Histoplasma capsulatum*, 293.4 MPHN) and one bacteria mcfDNA (*Escherichia coli,* 1081.7 MPHN) were detected in the patient's plasma sample. Regarding the three fungal detections, non-overlapping sets of mcfDNA reads aligned nearly perfectly and with uniform coverage to the genome assemblies of these respective fungi. Unlike other tests that are based on conserved genes, in this whole-genome diagnostic test the risk of cross-reactivity between these fungi is very low as they are not closely related and their whole-genome sequence similarity is low. This is consistent with the lack of overlap between mcfDNA reads aligning to these fungi. All three fungal detections were well above reporting thresholds.

She was started on trimethoprim-sulfamethoxazole for *PJP* but was transitioned to primaquine and clindamycin due to leukopenia secondary to trimethoprim-sulfamethoxazole, to complete a 21-day course of therapy. She was given intravenous liposomal amphotericin B 5mg/kg/day to treat both pulmonary histoplasmosis and coccidioidomycosis for 26 days followed by itraconazole thereafter. She was switched from itraconazole to isavuconazole for 39 days due to a transaminitis thought to be attributed to itraconazole, then transitioned back to itraconazole when her liver enzymes normalized. At the time, she had an AST of 188 (reference range 7–35 U/L), ALT of 272 (8–40 U/L), alkaline phosphatase of 155 (reference range 25–100 U/L), and total bilirubin of 0.3 (reference range 0.3–1.2 mg/dL). She was given dapsone for *PJP* prophylaxis following treatment. She completed therapy for her three opportunistic infections and agreed to start bictegravir/emtricitabine/tenofovir alafenamide at the time of her clinic follow-up visit.

## 4 Discussion

According to the CDC, fungal infections are responsible for over 75,000 hospital stays and almost 9 million clinic visits on a yearly basis [11]. The most frequently encountered fungal infections involving the lungs include aspergillosis, cryptococcosis, and *Pneumocystis jirovecii* [12]. Coccidioidomycosis and histoplasmosis are common in endemic regions. Most mixed fungal infections occur in immunocompromised patients, and mixed mycoses are often comprised of *Aspergillus* and *Cryptococcus* [12]. There have been studies involving *Aspergillus* and *Mucor* co-infection, and the majority of *Cryptococcus* and *Talaromyces marneffei* co-infections are noted in patients who have HIV [12]. Our case is particularly notable due to the rare concurrence of three distinct fungal infections identified in an immunocompromised host. The patient's markedly low CD4 count, indicative of advanced HIV infection, significantly increased her susceptibility to opportunistic pathogens. Additionally, residing in an endemic region likely contributed to the acquisition of these infections.

In one study conducted on 11 patients with HIV, combinations of Candida species were noted in the same patient: *Candida albicans* and *Candida tropicalis* were noted in 1 patient, *Candida albicans*, *Candida guilliermondii*, *Candida tropicalis*, and *Candida glabrata* were noted in a second patient, and *Candida albicans* and *Candida glabrata* were noted in a third patient [13]. Mixed-species fungal infections, including disseminated infections, can occur more commonly in the setting of immunocompromising conditions [13]. In particular, among severely immunocompromised patients such as transplant recipients and advanced HIV patients, mixed-fungal species infections can include species that typically colonize the body in the commensal state [13].

One retrospective single-center study revealed the importance of maintaining mixed mycosis on the differential in instances in which inadequate response to the original antifungal treatment occurs [12]. That study enrolled 17 patients with mixed mycoses between the years of 2011 and 2019 [12]. In that cohort, 8 patients were diagnosed with coinfection involving *Mucor* and *Aspergillus*, 4 patients had infection due to *Cryptococcus* and *Aspergillus*, 2 cases were from *Talaromyces marneffei* and *Cryptococcus*, 2 cases had *Talaromyces marneffei* and *Aspergillus*, and 1 case had *Candida* and *Aspergillus* [12]. *Aspergillus* and *Mucor* accounted for the majority of mixed fungal infections in this study [12].

Another retrospective study performed by Dr. Ramirez at a tertiary care hospital in Colombia evaluated patients with hematological cancers who developed invasive fungal infections over a 10-year time frame, and 22 cases of mixed fungal infections were found within this cohort [14]. In this study, 90 % of patients with mixed fungal infections were not only immunocompromised secondary to hematological malignancies, but they also experienced prolonged neutropenia [14]. The most frequently observed clinical syndromes in mixed fungal infections within this study included invasive pulmonary fungal infection and fungemia [14]. The most common combinations of mixed fungal infections within the context of this study were aspergillosis accompanied by invasive candidiasis (in 7 of the 22 cases), aspergillosis combined with fusariosis (in 3 of the 22 cases), and aspergillosis associated with *Curvularia* infection (in 3 of the 22 cases) [14].

Not only can mixed fungal infections involve mycoses from different genera, but they can also involve multiple strains of the same species [15]. According to one study, mixed infections of *Cryptococcus neoformans* can result from environmental exposures, but also from *in vivo* evolution resulting in endoreplication of the yeasts within the patient [15]. Exposure to strains of *Cryptococcus neoformans* comprised of varying genotypes or serotypes from the same geographic site can lead to mixed infections as well [15]. That study evaluated 100 isolates of *Cryptococcus neoformans* from 49 patients [15]. Mixed infections were witnessed in 9 of the 49 patients [15]. Serotypes A and D of *Cryptococcus neoformans*, genetically unrelated cultures of *Cryptococcus neoformans*, and hybrid strains of *Cryptococcus neoformans* were even observed in the same host [15].

In instances in which mixed fungal infection involves different genera infecting the same host, the clinical syndrome and chest imaging findings may not present as expected and create a diagnostic challenge [12]. When implicated in mixed fungal infection, an early halo sign or late crescent sign may not be observed on chest imaging in the setting of pulmonary aspergillosis, and a reverse halo sign might not be present in the setting of mucormycosis [12]. Thus, histopathology and culture play an important role in the identification of mixed mycoses [12]. In this case, the Karius test helped solidify the diagnosis of a mixed fungal infection earlier to facilitate treatment optimization in our immunocompromised patient.

Suspicion for mixed fungal infections can impact diagnostic and treatment decisions for a given patient. Careful analysis of the patient's history, clinical syndrome, fungal markers, cultures, histopathology, and imaging characteristics is necessary when determining if a patient has a mixed mycosis. The challenge that arises is that mixed mycoses infecting the same immunocompromised host can manifest with atypical presentations in terms of both clinical symptoms and radiographic findings. A Karius test can serve as an adjunct diagnostic test in this setting, because it can support other clinical and diagnostic criteria associated with each of the infections implicated in a mixed mycotic infection. The rise in the incidence of mixed mycoses can lead to treatment challenges. In patients with a presentation suspicious for a mixed mycotic infection, while gathering laboratory data to support each fungal infection, patients need to be started on broad empiric antimicrobials that can carry toxicities. Physicians can streamline treatment decisions if they have a positive Karius test early in an immunocompromised patient with an already high suspicion for mixed mycotic infection based on clinical, epidemiologic, and/or imaging factors. It will be important to pursue further research on which threshold values for a particular pathogen identified on a Karius test correspond to the clinical likelihood of that disease impacting a particular host.

In addition to noting the benefit of the Karius test in assisting with the diagnosis of this mixed fungal infection in an immunocompromised host, it is important to recognize the changing geographic distribution of certain fungi [8]. Histoplasmosis, coccidioidomycosis, and blastomycosis represent endemic mycoses [8]. The original geographic distributions outlined in the literature were made in accordance with antigen skin testing (for histoplasmosis and coccidioidomycosis) and case reports as well as outbreak reports (for blastomycosis) [8]. However, over the past half century, these three fungal infections have spread beyond their previously described geographic distributions resulting from factors such as climate change and other environmental effects [8]. A retrospective study collected data from 2007 to 2016 on 79,749 patients with histoplasmosis ≥65 years old [16]. Some regions that had cases of histoplasmosis included areas where it was not thought to be endemic including Arizona, California, Montana, Alaska, Washington, and Hawaii [16]. While histoplasmosis is more commonly observed in North America, South America, Central America, and parts of Africa, patients with a compatible clinical syndrome and imaging findings should be worked up appropriately [16]. Plans to increase access to antigen detection tests may improve the frequency of diagnosis in locations that are not typically characterized as histoplasmosis-endemic regions [16]. It remains possible that this patient acquired histoplasmosis while living in Virginia years ago, and that the infection reactivated after she moved to California while she was immunocompromised in the setting of advanced HIV [17]. Overall, it is very rare to see histoplasmosis in regions on the West Coast such as California, and according to data from the CDC in 2019, histoplasmosis was deemed a reportable condition in 13 states: Arkansas, Delaware, Illinois, Indiana, Kansas, Kentucky, Louisiana, Michigan, Minnesota, Nebraska, Pennsylvania, Rhode Island, and Wisconsin [17]. It remains to be explored how profound a change the geographic distribution of endemic mycoses will experience in the future due to factors such as climate change and other environmental factors.

The fact that all 3 fungal detections were above reporting thresholds facilitated the diagnosis in conjunction with the clinical syndrome, fungal serologies, and imaging noted in the workup. The positive streptococcal urine antigen test was likely a false positive in this clinical case, as the clinical syndrome, lack of response to antibiotics, and imaging findings were more characteristic of a fungal infection. The 2019 IDSA/ATS guidelines advise against routine use of pneumococcal urine antigen testing in adults with community-acquired pneumonia due to insufficient evidence that it ameliorates treatment outcomes [18]. Our patient did not have a high pretest probability of having pneumococcal pneumonia, and the likelihood of fungal infection was much higher given the progression of symptoms on antibiotic therapy and the time course of those symptoms. The aspergillus galactomannan antigen from the BAL was also likely a false positive. While the aspergillus galactomannan Ag from the BAL was 2.30, the diagnosis of aspergillosis was not corroborated by the imaging findings which showed nodular opacities. Radiographic patterns that can be encountered in pulmonary aspergillosis include lung cavities, air crescent sign, dense well-circumscribed lesion(s) with or without the presence of a halo sign, or wedge-shaped and segmental or lobar consolidation [19]. Additionally aspergillus species were not isolated on cultures and she had a negative aspergillus serum antigen test. Other fungi including *Histoplasma capsulatum* can have cross reactivity with aspergillus species. Aspergillus species were not detected by the Karius test as well. A respiratory pathogen panel was negative, thus there was low suspicion for a viral infection with a superimposed bacterial infection, but rather an evolving mixed mycosis. The negative COVID test also ruled out the possibility of a post-COVID aspergillosis. Our patient's severely immunocompromised status and lack of sufficient improvement with empiric antibiotics followed by *PJP* treatment raised concern for mixed mycotic infection. Patients with mixed fungal infections, especially if they are invasive, can decompensate rapidly if not treated with appropriate therapy. The positive Karius test for 3 fungal infections expedited the diagnosis and allowed for earlier treatment to enhance the patient's survival outcome. The patient clinically improved to the point of being able to follow up in clinic to start antiretroviral therapy. Additionally, even if the patient acquired the histoplasmosis infection in Virginia years ago and experienced reactivation in the setting of immunosuppression due to advanced HIV, this case rases awareness that the geographic distribution of endemic mycoses is slowly expanding.

## 5 Conclusion

This case expands our understanding of the presentation of mixed mycotic infections. It underscores the importance of broadening the differential diagnosis when an immunocompromised host does not respond to guideline directed therapy. Mixed mycoses can have atypical presentations in an immunocompromised host, thus histopathology and culture are frequently utilized to solidify the diagnosis [12]. However, in severely ill immunocompromised patients, a Karius test can be useful in expediting the diagnosis and treatment of mixed mycoses, thereby improving survival outcomes. Additionally, the geographic distribution of endemic mycoses is slowly changing. This patient may have originally acquired histoplasmosis while in Virginia years ago and experienced reactivation when she became immunocompromised in the setting of advanced HIV. However, climate change and other environmental factors have begun to slowly expand the geographic distribution of endemic mycoses, and this may impact the workup for fungal infections in the future.

## CRediT authorship contribution statement

**Elise Hyser:** Writing – review & editing, Writing – original draft, Conceptualization. **Shirisha Pasula:** Writing – review & editing, Supervision.

## Ethical statement

We have no conflicts of interest to disclose. No patient identifiable information was used in this case report.

## Conflict of interest statement

We have no conflicts of interest to disclose.

## References

[1] Clinicalinfo.HIV.gov (2025). Adult and adolescent opportunistic infection guidelines- Pneumocystis pneumonia. https://clinicalinfo.hiv.gov/sites/default/files/guidelines/documents/adult-adolescent-oi/pneumocystis-pneumonia-adult-adolescent-oi.pdf.

[2] Bennett J.E., Dolin R., Blaser M.J., Mandell (2020). Douglas, and Bennett’s principles and practice of infectious diseases.

[3] Clinicalinfo.HIV.gov (2025). Guidelines for the prevention and treatment of opportunistic infections in adults and adolescents with HIV: histoplasmosis. https://clinicalinfo.hiv.gov/en/guidelines/hiv-clinical-guidelines-adult-and-adolescent-opportunistic-infections/histoplasmosis.

[4] Knox K.S., Histoplasmosis C.A. Hage (2009). Proc. Am. Thorac. Soc..

[5] Barros N., Wheat J.L., Hage C. (2023). Pulmonary histoplasmosis: a clinical update. J. Fungi.

[6] Williams S.L., Chiller T. (2022). Update on the epidemiology, diagnosis, and treatment of coccidioidomycosis. J. Fungi.

[7] Sivasubramanian G., Kadakia S., Kim J.M., Pervaiz S., Yan Y., Libke R. (2023). Challenges in the long-term management of patients with coccidioidal meningitis: a retrospective analysis of treatment and outcomes. Open Forum Infect. Dis..

[8] Mazi P.B., Sahrmann J.M., Olsen M.A., Coler-Reilly A., Rauseo A.M., Pullen M., Zuniga-Moya J.C., Powderly W.G., Spec A. (2022). The geographic distribution of dimorphic mycoses in the United States for the modern era. Clin. Infect. Dis..

[9] Blauwkamp T.A., Thair S., Rosen M.J. (2019). Analytical and clinical validation of a microbial cell-free DNA sequencing test for infectious disease. Nat. Microbiol..

[10] Park S.Y., Chang E.J., Ledeboer N. (2023). Plasma microbial cell-free DNA sequencing from over 15,000 patients identified a broad spectrum of pathogens. J. Clin. Microbiol..

[11] CDC: Fungal Diseases (2024). Data and statistics on fungal diseases. https://www.cdc.gov/fungal/data-research/facts-stats/index.html.

[12] Zhan Y., Lu C., Li S., Zhao J., Li Z., Gu Y., Ye F. (2022). Successful management of mixed mycosis in HIV-negative patients with different immune status: a case series report. Front. Cell. Infect. Microbiol..

[13] Soll D.R., Brogden K.A., Guthmiller J.M. (2002). Polymicrobial Diseases.

[14] Ramirez I.C. (2025). Mixed fungal infection in patients with hematological malignancies. OFID.

[15] Desnos-Ollivier M., Spaulding S. Patel A.R., Charlier C., Garcia-Hermoso D., Nielsen K., Dromer F. (2010). Mixed fungal infections and *in vivo* evolution in the human fungal pathogen *Cryptococcus neoformans*. mBio.

[16] Schmidt T.E., Vieceli T., Damasceno L.S., Kimuda S., Pasqualotto A.C., Bahr N.C. (2025). Evolving epidemiology, improving diagnostic tests, and their importance for the correct diagnosis of histoplasmosis. J. Fungi.

[17] Smith D.J., Williams S.L., Benedict K.M., Jackson B.R., Toda M. (2022). Surveillance for coccidioidomycosis, histoplasmosis, and blastomycosis- United States, 2019. Center for Disease Control and Prevention Morbidity and Mortality Weekly Report.

[18] Metlay J.P., Waterer G.W., Long A.C., Anzueto A., Brozek J., Crothers K. (2019). ATS/IDSA guidelines for diagnosis and treatment of adults with community-acquired pneumonia. Am. J. Respir. Crit. Care Med..

[19] Alexander B.D., Lamoth F., Heussel C.P., Prokop C.S., Desai S.R., Morrissey C.O. (2021). Guidance on imaging for invasive pulmonary aspergillosis and mucormycosis: from the imaging working group for the revision and update of the consensus definitions of fungal disease from the EORTC/MSGERC. Clin. Infect. Dis..

